# Imaging Lung Function in Mice Using SPECT/CT and Per-Voxel Analysis

**DOI:** 10.1371/journal.pone.0042187

**Published:** 2012-08-03

**Authors:** Brian N. Jobse, Rod G. Rhem, Cory A. J. R. McCurry, Iris Q. Wang, N. Renée Labiris

**Affiliations:** 1 Department of Medicine, Division of Respirology, McMaster University, Hamilton, Ontario, Canada; 2 Department of Pathology and Molecular Medicine, Division of Respiratory Diseases and Allergy, McMaster Immunology Research Centre, McMaster University, Hamilton, Canada; 3 Firestone Institute for Respiratory Health, St. Joseph’s Healthcare, Hamilton, Canada; The University of Chicago, United States of America

## Abstract

Chronic lung disease is a major worldwide health concern but better tools are required to understand the underlying pathologies. Ventilation/perfusion (V/Q) single photon emission computed tomography (SPECT) with per-voxel analysis allows for non-invasive measurement of regional lung function. A clinically adapted V/Q methodology was used in healthy mice to investigate V/Q relationships. Twelve week-old mice were imaged to describe normal lung function while 36 week-old mice were imaged to determine how age affects V/Q. Mice were ventilated with Technegas™ and injected with ^99m^Tc-macroaggregated albumin to trace ventilation and perfusion, respectively. For both processes, SPECT and CT images were acquired, co-registered, and quantitatively analyzed. On a per-voxel basis, ventilation and perfusion were moderately correlated (R = 0.58±0.03) in 12 week old animals and a mean log(V/Q) ratio of −0.07±0.01 and standard deviation of 0.36±0.02 were found, defining the extent of V/Q matching. In contrast, 36 week old animals had significantly increased levels of V/Q mismatching throughout the periphery of the lung. Measures of V/Q were consistent across healthy animals and differences were observed with age demonstrating the capability of this technique in quantifying lung function. Per-voxel analysis and the ability to non-invasively assess lung function will aid in the investigation of chronic lung disease models and drug efficacy studies.

## Introduction

Chronic respiratory diseases such as asthma and chronic obstructive pulmonary disease (COPD) are burdensome on healthcare systems and are associated with substantial morbidity and mortality worldwide [1]. Methodologies for three-dimensional imaging of the lung are important as they allow for better characterization and understanding of these obstructive diseases [2]. While volumetric CT densitometry is now widely used, it generally contributes only structural information while other imaging techniques can provide functional information such as the contribution of alveolar ventilation (V) and pulmonary capillary perfusion (Q) to gas exchange within the lung [3]. There are non-imaging methods of V/Q assessment [4], and the forced-expiration pulmonary function tests (PFTs) currently used for diagnosis of obstructive diseases are relatively simple to perform, but these methods cannot evaluate lung function in a regional manner. In addition, PFTs are unable to differentiate pathologies and are insensitive to early disease [5]. While imaging methods are technically more difficult than PFTs they can provide both global and regional functional information. Further, these volumetric techniques are necessary to address the heterogeneous underlying pathologies that make up diseases such as COPD [2], [6], [7].

In the field of clinical nuclear medicine, static V/Q imaging is routinely performed with single photon emission computed tomography (SPECT) along with Technegas™ and ^99m^Techetium-labelled macroaggregated albumin (MAA) to provide the distributions of V and Q, respectively. Images acquired by this method depict the relative contributions of the core pulmonary processes of V and Q within any particular region in the lung. When V/Q images are co-registered to CT, regional analysis can be coupled to structural information. Clinical research examining the pathophysiology of COPD has been shown to benefit significantly from the use of radiologist-scored V/Q SPECT/CT [6] but investigation into the use of per-voxel volumetric analysis has only just begun in this area [8], [9].

Pre-clinical models illustrating the utility of SPECT/CT as a tool in respiratory research are currently scarce [10]. Non-invasive assessment of small animal models offers an approach to address a natural link between discoveries at the molecular level and the application of clinically relevant diagnostics or therapeutics [10], [11], [12]. 3D imaging has now been used in various pulmonary disease models including asthma and emphysema, among others [10], [13], [14]. In regards to preclinical functional lung imaging, V/Q scanning has only seen service in rats through use of magnetic resonance imaging (MRI) and has so far lacked whole lung assessment [15], [16]. Global lung measurements of V/Q have also been made in rats using the multiple inert gas elimination technique [17], but as this methodology does not employ any imaging modality it cannot offer regional analysis. Although rats offer better resolution of lung structure due to their size, the ability to test molecular hypotheses is greater in mice due to the relative abundance of knockout strains and commercially available tests. The mouse has yet to be investigated by any V/Q methodology and no extensive quantitative V/Q evaluation of lung function has been performed in a murine model.

Utilizing V/Q SPECT/CT to measure functional consequences of known pathologies in murine models could improve our understanding of the pathophysiology of respiratory disease and translate knowledge gained to the clinical setting, where similar imaging methodologies can be applied in most nuclear medicine departments. In this study we have successfully adapted a common clinical V/Q imaging protocol, employing Technegas™ and ^99m^Tc-MAA, to mice. In addition, we have applied a three-dimensional quantitative per-voxel analysis to characterize the naïve lung environment. Further, the sensitivity of this lung function measurement was tested by global and regional assessment of age-related changes. This work provides a foundation for further investigation of lung function in respiratory disease models.

## Materials & Methods

### Animals

Female BALB/c mice (Charles River, QC, Canada) were acclimatised to specific pathogen-free housing conditions for a period of 2–3 weeks, with a 12∶12 hour light:dark cycle, prior to imaging. Experimentation was performed on 17 mice (12–16 weeks old, henceforth referred to as 12 w.o.) while another group of mice were housed for a further 24 weeks before imaging (henceforth referred to as 36 w.o.). The experiments described in this study were approved by the Animal Research Ethics Board of McMaster University (Animal Utilization Protocol #080514) in accordance with the guidelines of the Canadian Council on Animal Care.

### Imaging Protocol

Healthy, untreated mice were tracheally intubated through the oral cavity with a 20-gauge catheter following anaesthetisation with ketamine/xylazine (90 mg/kg, 6 mg/kg). Animals were then ventilated (0.02 L/min, 125 strokes/min) on a Rodent Ventilator (Model 683, Harvard Apparatus, Holliston, MA, USA) with Technegas™ (Cyclomedica, Lucas Heights, NSW, Australia) (0.04–0.12 MBq/mL) for 15 minutes, alternating between Technegas™ and room air in intervals of 15–30 seconds. Technegas™ is an ultrafine aerosol composed of carbon particles labelled with ^99m^Tc and suspended in argon, with a mean aerosol diameter between 30 and 60 nm [18]. Following ventilation mice were moved and strapped securely to a heated bed for animal welfare and consistency in body temperature between animals. SPECT scans were acquired on an X-SPECT system (Gamma Medica, Northridge, CA, USA) using dual sodium iodide crystals in combination with low energy pinhole collimators with 1 mm aperture and a radius of rotation of 3.5 cm. The ventilation SPECT scan consisted of thirty-two 50 second projections and was followed immediately by the collection of four rotations of 1024 X-ray projections for CT, also acquired on the X-SPECT system with x-ray tube characteristics of 75kVp and 220 µA. Following ventilation SPECT and CT scans, mice were injected with 11–15 MBq of ^99m^Tc-MAA via the tail vein. Care was taken not to shift the position of the animal during the injection. Perfusion imaging entailed a 1024-projection CT scan and a SPECT scan of thirty-two 40 second projections. Supplemental gaseous anaesthesia was used (isoflurane (1%, 1 L/min)) to ensure sedation throughout the imaging procedure (approximately 1 hour 20 minutes).

**Figure 1:**
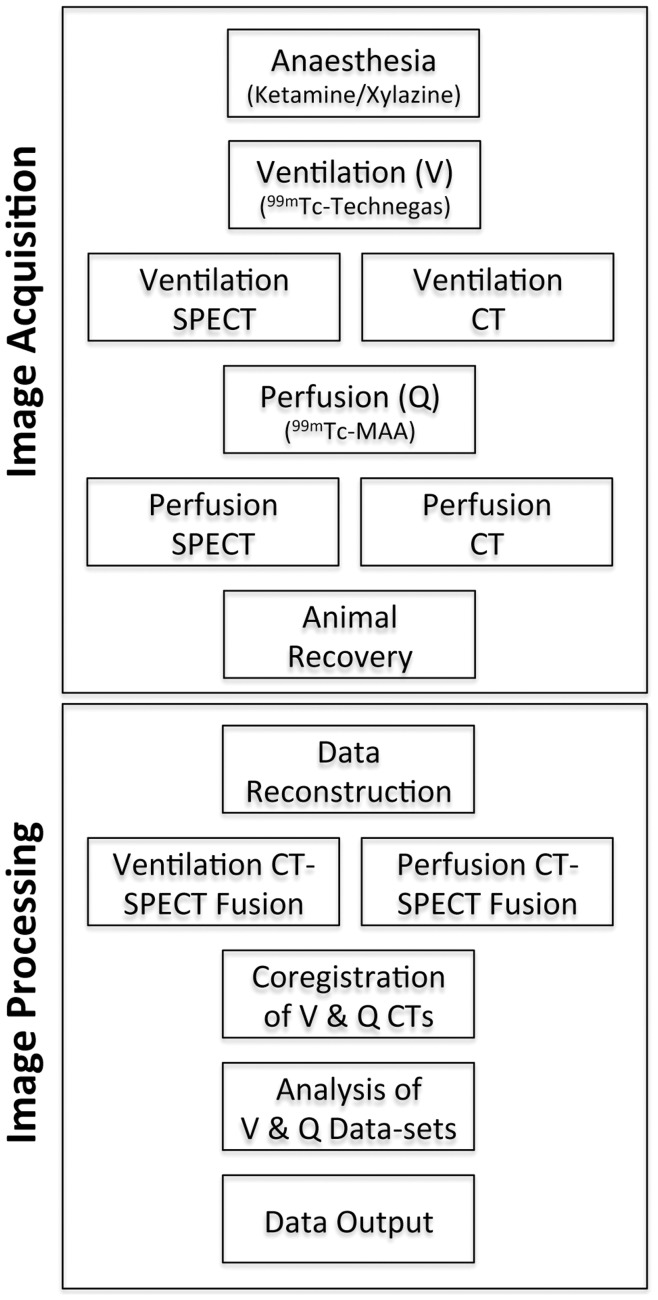
Simplified representation of the methodology used in image acquisition and processing to provide the final V/Q data sets.

Mice were recovered from the imaging procedure and monitored until radioactivity had decayed to background levels. All imaging work was completed at the McMaster Centre for Preclinical and Translational Imaging (MCPTI) at McMaster University (Hamilton, ON, Canada). A simplified protocol, detailing the sequence of imaging methodology, is shown in Figure 1.

### Reconstruction of 3D SPECT/CT Images

SPECT images for both ventilation and perfusion scans were produced by iterative reconstruction of their respective projections using FLEX™-SPECT software (Gamma Medica) into 80×80×80 arrays (0.472 mm isotropic voxels). The projections from the four ventilation CT rotations were first summed, then images were reconstructed using a Feldkamp cone beam backprojection algorithm in COBRA (Exxim Sofware, Pleasanton, CA, USA) into 512×512×512 arrays (0.115 mm isotropic voxels). This high quality CT image was used in the processing and analysis of V/Q data to provide anatomical features for SPECT co-registration and to also allow for a density-based assessment of the lung environment. The perfusion CT projections were reconstructed under the same conditions. Calibration of each CT image for Hounsfield Unit (HU) scaling was performed using empty airspace within the field of view and a water-filled tube included in each scan.

### Fusion and Co-registration of Ventilation and Perfusion SPECT/CT Data

**Figure 2:**
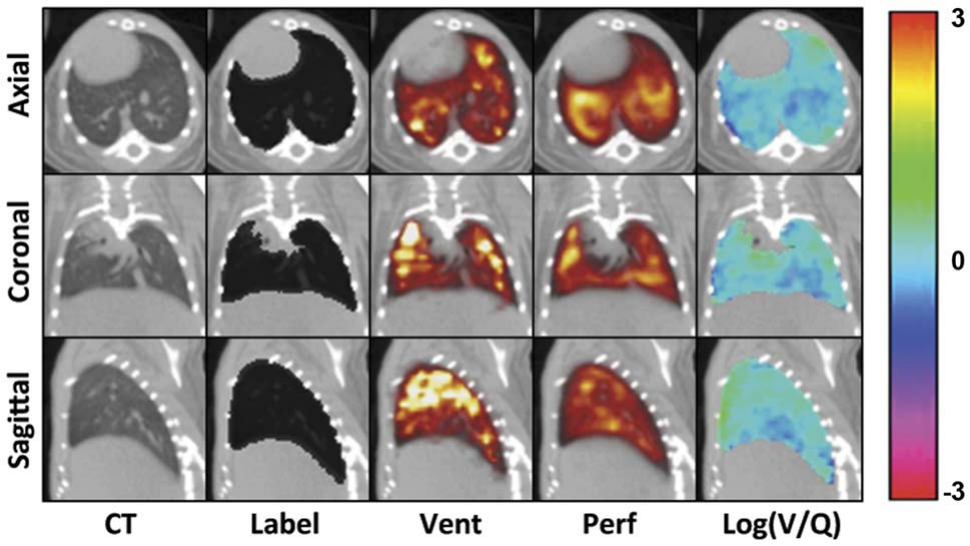
Representative CT and SPECT slice images in axial, coronal, and sagittal planes. The animal shown was chosen based on average mean and standard deviation values for the data set. **CT** refers to the ventilation-associated CT. **Label** refers to the lung segmentation seen as a black overlay on the CT. **Vent** and **Perf** refer to V and Q images with the colour scale increasing from red through yellow to white for both. **Log(V/Q)** refers to the final calculated data and the colour bar indicates the values seen in the associated log(V/Q) images.

**Figure 3:**
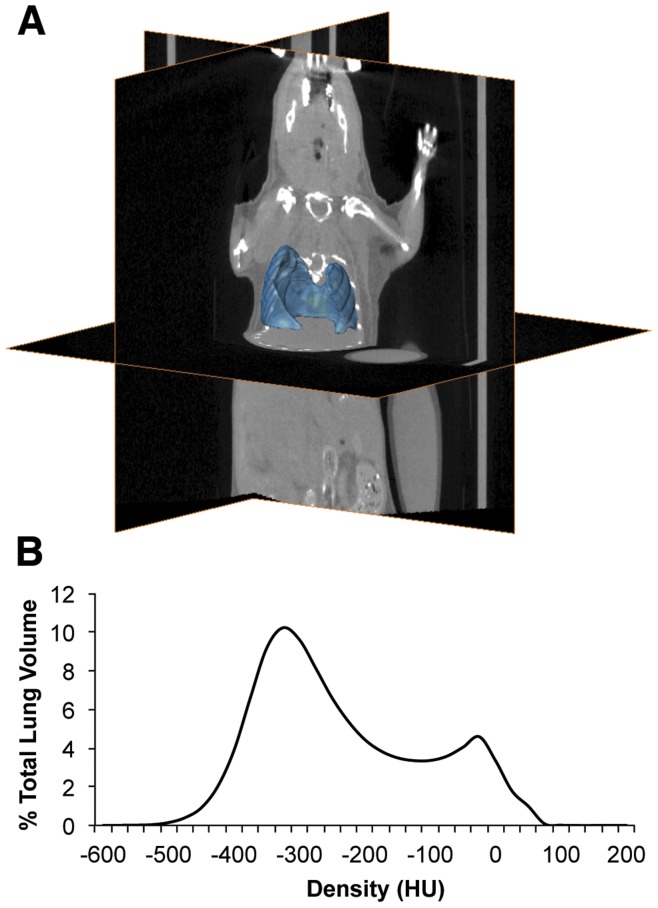
Lung segmentation used in the V/Q analysis. **A** Representative lung segmentation used in V/Q co-registration and analysis, with CT orthoslices for reference. **B** Average volume-standardized CT densitometry of ungated images in Hounsfield units (HU) depicting the distribution of densities within the label.

Ventilation and perfusion CT images (512^3^ matrix) were first subjected to a Gaussian filter (σ = 0.8) using Matlab version 7.5 (Mathworks, Natick, MA, USA). A ‘Lung’ label was produced for the CT images as described previously for rats [13] using Amira 5.1 software (Visage Imaging, Andover, MA, USA), with the exception that the trachea and main bronchi (up to the point where they enter lung tissue) were removed from the ventilation lung label. Briefly, a −50HU threshold-limited growing algorithm was used to define the airspace. This selection was then subjected to a simple volume-growing algorithm and another threshold of 100HU applied to exclude tissues and structures not belonging to the lung. A hole-filling algorithm was also applied to include internal structures such as major blood vessels and, finally, the label was manually examined to ensure the process was consistent across animals. CT images and lung labels were then compressed to a matrix of 256^3^ for computational purposes. Representative axial, coronal, and sagittal images for the CT, lung label, ventilation, and perfusion scans can be seen in Figure 2 while a representative lung label is shown in Figure 3A.

The perfusion CT and SPECT were fused, with in-house software developed in Matlab, by a process that maximised mutual information (MI) within the lung region as defined by the label field [19]. Powell’s multidimensional direction set method was used to maximise MI using a one dimensional search algorithm based on golden section search and parabolic interpolation [20]: SPECT images underwent rigid body transformation until a change of less than 0.01 mm (translation) or 0.01 degrees (rotation) was observed along or around each axis to obtain fusion parameters. During the process, the 80^3^ matrix of the SPECT image was interpolated for comparison to the CT image and was resampled to a 256^3^ matrix when the stop criteria were met; this corresponds to a change in voxel size from 0.40 mm^3^ to 0.23 mm^3^. When maximised MI was reached the result was visually inspected to confirm fusion quality. Parameters obtained from fusion of the perfusion SPECT and CT were then applied to the ventilation SPECT and CT as the spatial relationship between these two data sets remains constant.

The ventilation and perfusion data sets were then co-registered by repeating the MI maximisation process through rigid body transformation of their respective CT images, again within the lung segmentation. Specifically, the perfusion CT was adjusted to fit the ventilation CT. These co-registration parameters were then applied to the perfusion SPECT data allowing for comparison of ventilation and perfusion SPECT images. A simplified representation of image processing is shown in Figure 1.

### Quantitative Per-Voxel Assessment

Co-registration of images allows for analysis of SPECT and CT data on a per-voxel basis. Within the lung label all voxels had a HU, V, Q, and log(V/Q) value; presented data represent distributions of all voxels within the lung label averaged across all animals, unless otherwise stated. V and Q values were converted to relative frequencies by dividing the activity value of each voxel by the total activity in the lung label and then multiplying by 100. The relative frequency of both V and Q were used to calculate V/Q ratios and a base 10 logarithm was applied to provide a log-normal distribution. Analysis of the log(V/Q) data involved calculation of the mean, standard deviation, skewness, excess kurtosis, and percentage of total lung volume (TLV) greater than ±2 averaged standard deviations from the averaged mean log(V/Q), as a measure of V/Q mismatching. Any voxel where only V equalled zero was set to a log(V/Q) ratio of −∞ and any voxel where only Q equalled zero was set to a log(V/Q) ratio of +∞; these values were not included in the calculation of the log(V/Q) mean, standard deviation, skew or kurtosis. Any voxels where both V and Q equalled zero were noted and given a log(V/Q) value of zero.

### Regionalization of Images

Two different methods of regionalization were performed on V, Q, and log(V/Q) data. Apex, middle, and base divisions were made by finding the axial slices closest to the 33 and 66 percentiles of volume. Inner and outer divisions were made by eroding the outer boundary of the lung label by 3 voxels, using a six-neighbour structuring element in a volumetric manner, and subtracting the new inner data from the original whole lung to produce the outer data. Standard deviations of log(V/Q) were calculated for all voxels in a region in the same manner as described above.

### Respiratory-gated CT Image Processing & Quantitation

Inspiratory-and expiratory-gated CT images were produced by applying RespGate software (RespGate, Hamilton, ON, Canada) [21] to all ventilation CT projections. Respiratory-gated images were then reconstructed and calibrated as described for the ungated image used in the V/Q analysis. Air volume information from respiratory-gated CTs was extracted by a method previously described in rats [13]. Briefly, a segmentation was made encompassing the thoracic cavity using Amira 5.1 software and a histogram of the densities (25HU/bin) within this region of interest was produced. The volume of air within the thoracic segmentation was calculated from the histogram by multiplying the number of voxels in a HU density bin against an air coefficient for that HU bin and converting the result into millilitres. The air coefficient was defined by the centre of the HU bin and represented the fraction of air in a voxel of that value; e.g., the air coefficient for −500 HU is 0.5. Summation of the air-histogram from the end expiratory image allowed for quantitation of functional residual capacity (FRC) and tidal volume (TV) was calculated as the difference between air volume in the inspiratory and expiratory lung states.

### Data Analysis

Images were analyzed and all measures were output with the aid of Matlab. Boxplots represent the lower quartile, median and upper quartile while whiskers represent the minimum and maximum values within the data set. Data are expressed as the mean±SEM and, where applicable, statistical significance was determined by unpaired two-tailed t-test in Graphpad Prism (Graphpad Software Inc, La Jolla, CA, USA); p<0.05 was considered statistically significant. Two of the seventeen 12 w.o. animals were excluded for the following reasons: one V/Q data set was a statistical outlier for the ratio of total perfusion activity to total ventilation activity, and another animal received an improper intravenous delivery of MAA, as noted at the time of injection. One 36 w.o. animal was also excluded as an outlier for increased FRC volume.

## Results

Many lung function parameters can be measured from V/Q SPECT/CT: Ventilation and perfusion distribution within the lung can be determined; the relationship between these processes can be studied by measuring their correlation on a per-voxel basis; the degree of V/Q mismatching is illustrated in the log(V/Q) distribution and can be quantified using thresholds, such as two standard deviations from the mean; from the CT, tidal volume and FRC can be quantified in addition to changes in lung density. Investigating these parameters provides a detailed evaluation from which lung dysfunction can be assessed.

Typically, the distribution of deposition in the ventilation images was somewhat heterogeneous and typified by areas of increased intensities, predominately in apical regions, while decreased intensities were observed in basal regions (Figure 2). The lung perfusion distribution demonstrated greater uniformity of counts with less apparent skew towards the upper regions of the lung. Similarly, log(V/Q) images described a relatively homogeneous distribution with only small regions of obvious V/Q mismatch. The average total number of counts from ventilation with Technegas™, measured within the lung, was 1.96±0.33×10^5^ while the average total number of counts from perfusion with ^99m^Tc-MAA was 3.61±0.31×10^6^ (data not shown) creating a mean ratio of 24.2±3.7 to 1 between perfusion and ventilation total activity. This ratio was unaltered in the 36w.o. animals.

**Figure 4:**
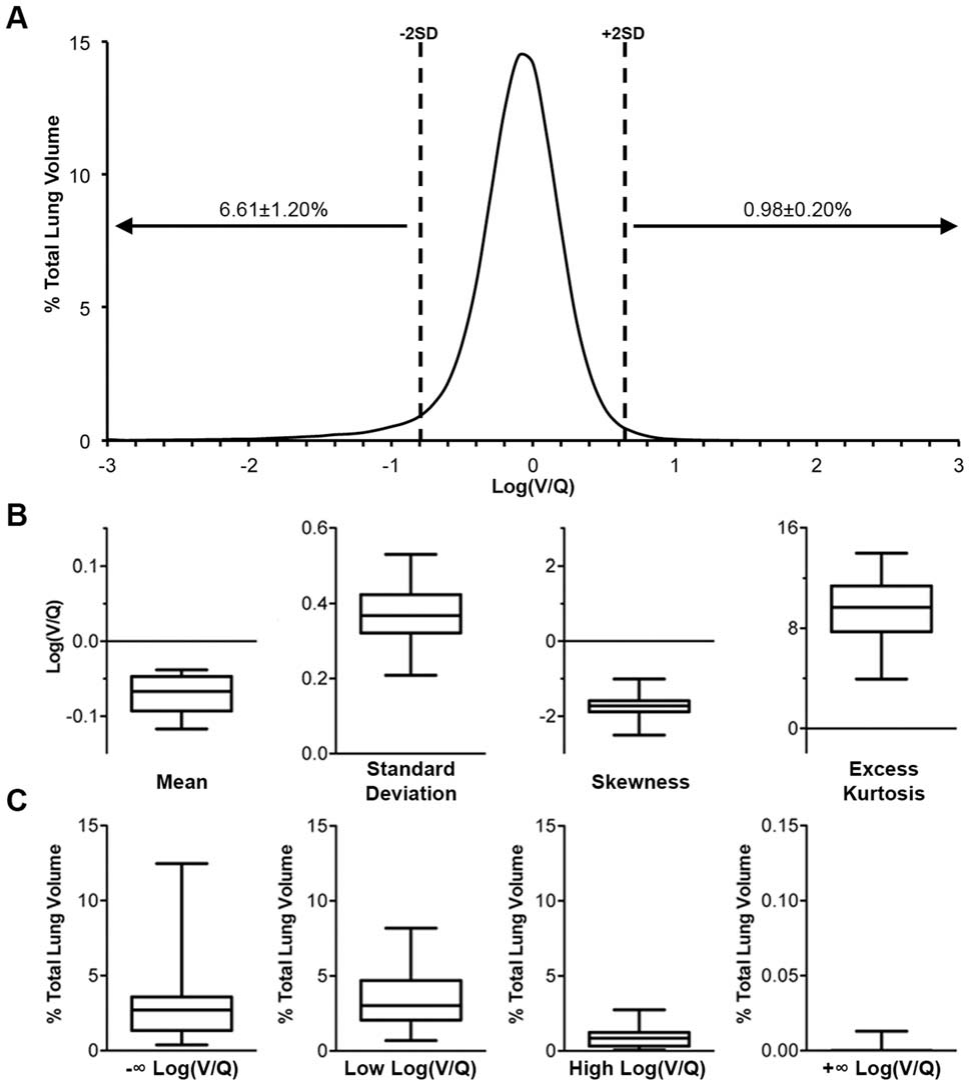
Distribution and measures of log(V/Q) data. **A** Average volume-standardized distribution of log(V/Q) values measured in percentage of total lung volume. Dashed lines indicate average ±2 standard deviations from the average mean. Values over arrows represent average±SEM percentage of lung volume beyond ±2 standard deviations, including log(V/Q) ±∞ values. **B** Descriptive analysis of log(V/Q) distribution central tendency and variation within the lung, calculated without ±∞ values. **C** Percentage of total lung volume with log(V/Q) mismatching. Mismatching is defined as values greater or less than 2 average standard deviations from the average mean. −∞ and +∞ refer to voxels where V = 0 and Q = 0, respectively. Box plots describe the data for all 12w.o. animals in terms of the median, interquartile range, and minimum/maximum values.

The degree of ventilation and perfusion matching in the lung was determined by plotting the distribution of log(V/Q) values (Figure 4A). The mean and standard deviation of log(V/Q) values were narrowly distributed with averages of −0.071±0.006 and 0.36±0.02, respectively (Figure 4A & B). In addition, the log(V/Q) distribution was not normal in nature as it was negatively skewed and positively kurtotic. Approximately 6.6% of TLV had a low log(V/Q) ratio mismatch while approximately 1.0% of TLV had a high log(V/Q) ratio mismatch (Figure 4A). Of the low log(V/Q) mismatched volume 3.23±0.75% of TLV was made up of voxels with a log(V/Q) value of −∞ but no significant proportion of voxels with values of +∞ were found (Figure 4C).

**Figure 5:**
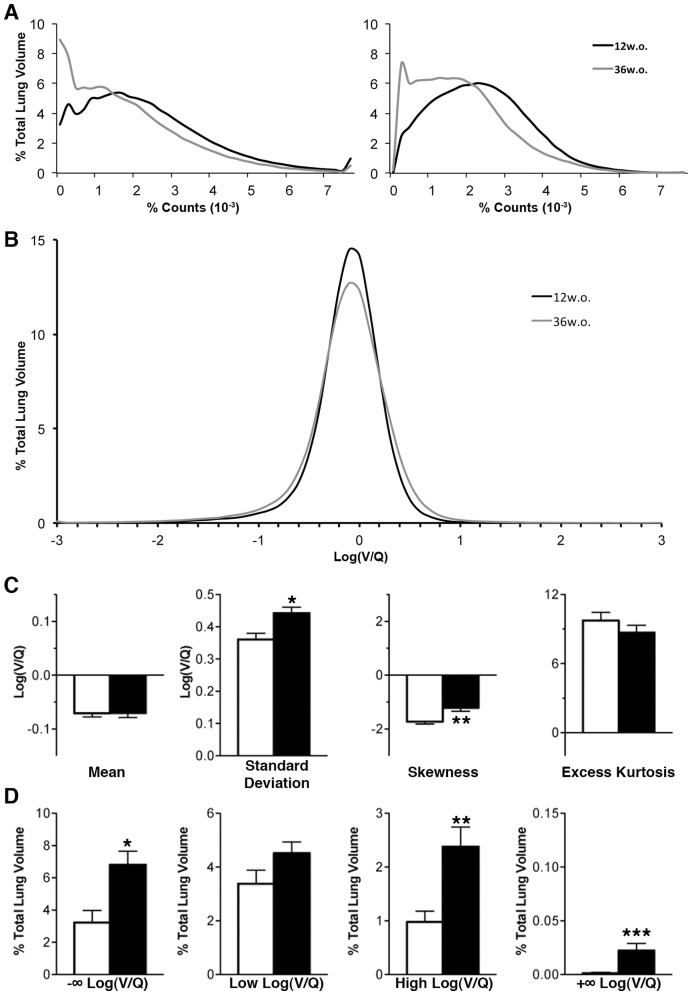
Effects of aging on lung function. **A** Average relative count distributions of ventilation (left) and perfusion (right) for 12w.o. (black) and 36w.o. (grey) animals in percent of total lung volume. **B** Average log(V/Q) distributions for 12w.o. (black) and 36w.o. (grey) animals. **C** Descriptive analysis of log(V/Q) distribution central tendency and variation within the lung, calculated without ±∞ values. **D** Percentage of total lung volume with log(V/Q) mismatching. Mismatching is defined as values greater or less than 2 average standard deviations from the average mean. −∞ and +∞ refer to voxels where V = 0 and Q = 0, respectively. White bars refer to averages and SEM for 12w.o. animals while black bars refer to averages and SEM 36w.o. animals. *p<0.05, **p<0.01 by two-tailed t-test.

In the comparison of 12w.o. to 36w.o. animals, discrete histograms were generated for both ventilation and perfusion in order to assess any difference due to age in these physiological processes; 36w.o. animals demonstrated a shift towards lower relative count values for both ventilation and perfusion when compared to their younger counterparts (Figure 5A). The leftward shift of these distributions was not apparent when these processes were translated to a per-voxel log(V/Q) ratio, where the difference between 12w.o. and 36w.o. animals is much more subtle (Figure 5B). However, older animals had significant changes in standard deviation, skewness, and excess kurtosis of the log(V/Q) distribution (Figure 5C). Furthermore, values for positive and negative ∞ were significantly increased along with values for high log(V/Q) (Figure 5D); these changes were larger in magnitude for negative values than positive.

**Figure 6:**
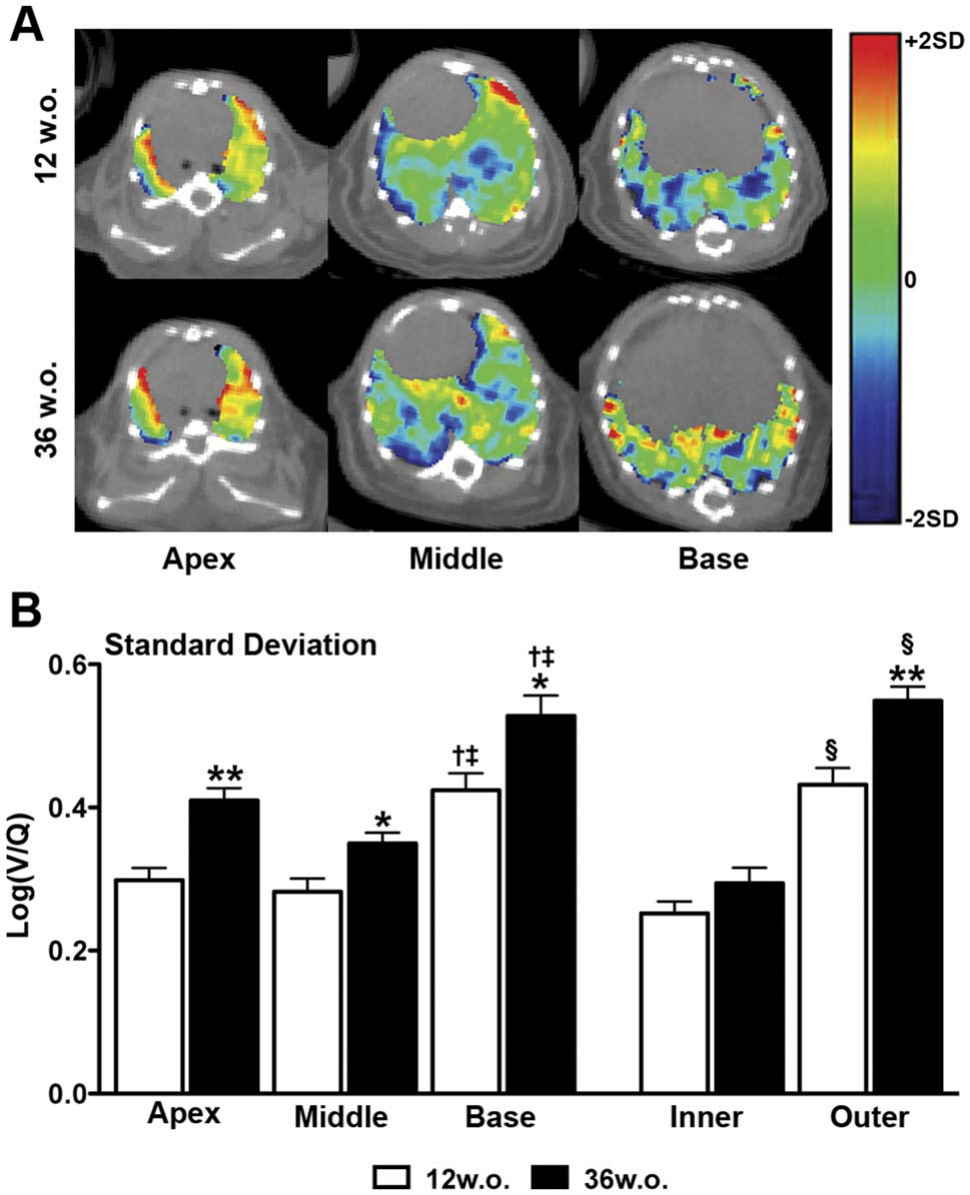
Regionalization of V/Q mismatching. **A** Representative axial log(V/Q) slices depicting changes with age in the apex, middle, and base of the lung. The colour scale represents 2 average standard deviations from the average mean of 12w.o. animals. **B** Regional analysis of averaged log(V/Q) standard deviation values in the apex, middle, and base of the lung as well as inner and outer regions. *p<0.05, **p<0.01 by two-tailed t-test between 12 and 36w.o. groups. **†**p<0.05 apex vs. base, **‡**p<0.05 middle vs. base by one-way ANOVA with Tukey post-hoc. **§**p<0.05 by two-tailed t-test between inner and outer regions.

Log(V/Q) images of 12 and 36w.o. animals indicated that V/Q mismatching was distributed throughout the lung (Figure 6A). Regionalization of lung volume into apex, middle, and base divisions (approximately 33.3% of volume per division) as well as inner and outer divisions (approximately 45 and 55% of volume per division, respectively) allowed for further analysis of the distribution of V/Q heterogeneity and mismatch; no change in regional volume proportion was observed with age. In 12w.o. animals, the standard deviation of log(V/Q) values was greater in the basal region of the lung compared to the apex or middle regions (Figure 6B). In 36w.o. animals increases in standard deviation over 12.w.o. values were observed in all three regions. Inner and outer regionalization demonstrated that the increase in standard deviation values with age was greater in the periphery of the lung (Figure 6B). This age-related difference in the outer region was associated with a significant decrease in the percentage of both ventilation and perfusion counts in that region (data not shown), indicating that the increase in V/Q heterogeneity is associated with alterations in the distribution of both processes. This regional distribution of increased log(V/Q) heterogeneity indicates that mismatching is present throughout the lung and is primarily associated with the periphery.

**Figure 7:**
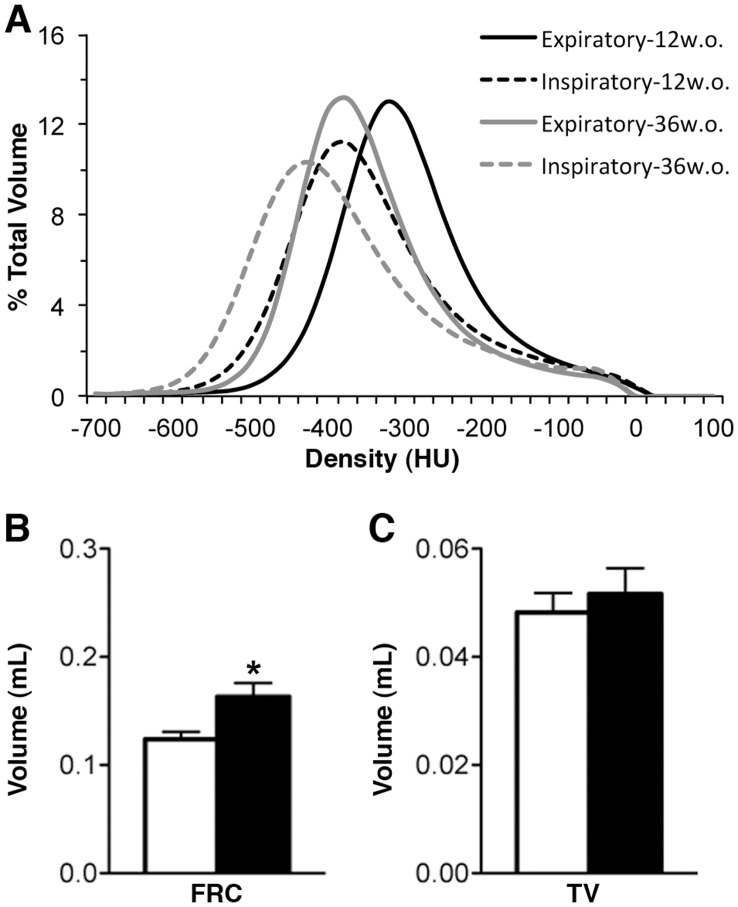
Respiratory-gated CT data for 12w.o. and 36w.o. groups. **A** Average volume standardized Hounsfield unit (HU) densitometry of expiratory (solid line) and inspiratory (dashed line) lung states for 12w.o. (black) and 36w.o. (grey) animals. **B** Average functional residual capacity (FRC) and tidal volume (TV), calculated from gated CT data. White bars refer to 12w.o. animals while black bars refer to 36w.o. animals. ***p<0.001 by two-tailed t-test.

The CT density distribution of the lung was described by a bimodal curve with a primary peak at approximately −325HU, describing the air volume, and a lesser peak at approximately −25HU, describing tissue volumes within or surrounding the lung (Figure 3B). Post-acquisition respiratory gating produced CT images of end inspiratory and end expiratory lung states. These curves are unimodal in nature due to the calculation of air content based on HU. There were notable differences in density distributions between inspiratory and expiratory states including a broadening of the peak and a shift towards lower HU values during inspiration (Figure 7A). These curves were further shifted towards lower densities in older mice. For 12w.o. animals, FRC was determined to be 0.12±0.01 mL while TV was determined to be 0.048±0.004 mL. FRC was significantly increased in the 36w.o. group while TV remained constant (Figure 7B).

When FRC, denoting lung volume, was plotted against the median value for ventilation and perfusion distributions a high degree of correlation (R^2^ of 0.77, p<0.0001 and 0.75, p<0.0001 respectively) was observed for all 12w.o. animals indicating that the leftward shift in these distributions in the 36w.o. animals was due to the increased FRC values in this group. However, no correlation was seen when FRC was plotted against the standard deviation of the log(V/Q) curve, as a general measure of V/Q heterogeneity, indicating that FRC is not a driving factor in the log(V/Q) mismatch observed in older animals. In addition to the increase in FRC, a small increase in body mass was observed in 36w.o. mice (Table 1).

**Table 1:** Comparison of group characteristics between 12 week old and 36 week old mice.

	12 w.o.	36 w.o.
Number	15	6
Mass (g)	22±0.3	24±0.6*
Q:V Ratio	24.2±3.7	14.2±1.7
Log(V/Q) Mean	−0.071±0.007	−0.071±0.008
Log(V/Q) Standard Deviation	0.361±0.020	0.442±0.018*
Log(V/Q) Skewness	−1.73±0.09	−1.22±0.13**
Log(V/Q) Excess Kurtosis	9.75±0.70	8.70±0.63
Log(V/Q) −∞ values	3.23±0.75	6.82±0.83*
Log(V/Q) +∞ values	0.0012±0.0009	0.0223±0.0066***
Log(V/Q) Low values	3.38±0.50	4.52±0.42
Log(V/Q) High values	0.98±0.20	2.38±0.36**
Functional Residual Capacity (mL)	0.124±0.007	0.163±0.013*
Tidal Volume (mL)	0.048±0.004	0.052±0.005

* p<0.05, **p<0.01, ***p<0.001 by two-tailed t-test against 12 w.o. animals.

## Discussion

The capability to study the core processes of a vital organ, namely ventilation and perfusion in the lung, at a scale relevant to these processes represents an imperative step towards understanding lung function and how it changes in respiratory disease. This study demonstrates that we have successfully translated a common clinical V/Q imaging technique to the preclinical setting to measure lung function in mice. Using V/Q SPECT/CT as a biomarker of lung function will support preclinical investigations of the pathogenesis of respiratory diseases and screening of novel drug therapies, helping to bridge the gap to the clinical understanding of disease and drug efficacy. In addition, we have developed a preclinical method of image co-registration and data analysis to objectively evaluate V/Q at a per-voxel level so that regional assessments of lung function can be performed.

A V/Q ratio of 0.80, or a log(V/Q) of −0.097, is considered optimal for a healthy human lung [22]. We found a mean log(V/Q) of −0.071±0.006, corresponding to a V/Q ratio of 0.85, in our naïve mice indicating that healthy mammalian lungs have similar respiratory physiology. These values represent the gas exchange capability for the entire lung, but ventilation and perfusion, at a small scale, are somewhat heterogeneous processes. While early work suggested that gravity was a large determinant of heterogeneity in V/Q ratios [23], methodologies with better resolution describe greater variability at any particular axial height within the lung for both ventilation and perfusion [24]. It is now thought that the majority of this intrinsic variability can be attributed to factors related to the basic architecture of the lung [25]. To define the natural degree of heterogeneity in this model V/Q mismatching was defined as the percentage of total lung volume (TLV) with log(V/Q) ratios ±2 standard deviations from the mean, as 95% of values should fall within this range for a normal distribution. For this purpose the average mean and standard deviation of the total data set was used with the notion that severely mismatched voxels would fall beyond the scope of this average “normally functioning” region. Only a small percentage of volume (∼1%) was found above +2SD while a higher proportion (∼7%) was found below −2SD. This population included voxels where ventilation values, but not perfusion values, equalled zero and thus had a log(V/Q) value of −∞. We found that approximately 3% of TLV had such a log(V/Q). Most of these zero ventilation values appear at the perimeter of the lung-label, especially around the caudal-most edges, and likely represent tissues not associated with alveolar regions or marginal errors in the co-registration process that were included in the lung label. Such voxels that are non-lung could contribute to the ±∞ categories; however, since the segmentation employed is a standardized process any sampling errors between animals should be consistent. Overall, the variability in V/Q distribution within the naïve animals studied was low, with a log(V/Q) standard deviation of 0.36±0.02, and therefore represents a typical degree of ventilation and perfusion heterogeneity in this system.

In humans, a decline in V/Q matching is known to occur with increased age [26], [27], [28] so we elected to study the effect of aging on lung function to determine the sensitivity of our methodology. To provide an experimental model to this effect a subset of mice were allowed to age for approximately 6 months, which is a considerable amount of time in the life of a mouse. Though the mice at 36 weeks of age had significantly more mass, and obesity is known to impact lung function [29], these mice were not considered obese and their weight would have little, if any, impact on V/Q matching (R = 0.1426, p = 0.79). However, we found that older animals had a significant increase in V/Q mismatching, as defined by the standard deviation of the log(V/Q) data, over their younger counterparts suggesting that this measure is sensitive to minor changes in lung function. The greater FRC observed in 36w.o. animals is likely associated with a reduction in lung elasticity, which is in turn due to a general loss of alveolar surface area [26]. This change in alveolar structure is potentially responsible for the increased V/Q heterogeneity demonstrated in older mice as similar mechanisms have been proposed clinically [30]. Regionalization of images further indicated that the increase in standard deviation of the log(V/Q) data is spread throughout the lung, but is also primarily associated with the peripheral volumes. The increase in standard deviation values in the outer region was associated with decreases in the percentages of both ventilation and perfusion associated with this volume. As greater than 30% of ventilation and perfusion counts were still present in this region at 36 weeks of age, and both ventilation and perfusion were decreased, it is unclear from this data what process is causing the increased V/Q mismatch observed. A decrease in perfusion with increasing distance toward the periphery has been noted previously by other methods [31] but the peripheral involvement of V/Q mismatch with age requires further research.

This study employed a per-voxel analysis in order to better depict the intrinsic V/Q heterogeneity in the lungs. Although the voxels used (0.23 mm^3^) were larger than the alveolar structure, a great number of V/Q measurements were made within the lung label, forty-six thousand on average for a mouse lung. The strength of this approach is that measurements of the whole lung, such as mean and standard deviation, can be broken down into constituent parts. Small areas of mismatched V/Q will not necessarily stand out in global measurements but even these regions, in a voxel-by-voxel analysis, are likely made up of many voxels. The location and extent of mismatching could be quantified and a better understanding gained of how the lung functions on a regional level. We elected to use a segmentation defined from the ungated CT to ensure that all lung-associated voxels within the ungated SPECT images were included in the analysis. Suga *et al.* [8], in a clinical study, employed a threshold of 10% of maximum in their ventilation image data to define a region of interest for the lungs, but this method fails to address any regions of low ventilation activity and therefore could potentially exclude regions of lung, depending on the disease or model being used. Inclusion of all lung-associated volume is preferable and anatomical imaging, such as CT, is therefore not only desirable but also necessary.

Compared to clinical V/Q SPECT, the method used to deliver Technegas™ had to be modified for this preclinical work; while human patients are capable of inspiring an entire dose of activity in one to three breaths, mice require mechanical ventilation over the course of several minutes with Technegas™ at a relatively high level of radioactivity. Anaesthetic use is a further difference, though we intentionally kept the doses of these agents at a minimum to decrease any possible effect on respiration; respiratory gating confirmed that breathing patterns were similar between animals. Despite the differences between a clinical V/Q and the preclinical technique demonstrated here the results of this study represent a comparable methodology that achieves consistent results.

An important consideration for this methodology is the radiation dose to the subject animal. Work by Travis *et al*. suggests that the lethal dose for 50% (LD50) for BALB/c mice is above 5Gy and that the dose rate required is well beyond that used in our study. Furthermore, no pathological changes were observed in the lungs or kidneys at a dose-rate of 100 mGy/min [32]. Based on previous whole body dosimetry measurements of mice using the same X-SPECT system, average CT radiation absorption dose can be approximated at 270 mGy [14], delivered over the course of approximately 25 minutes. Further, whole body dose from ^99m^Tc exposure can be approximated at 51 mGy [33]. Thus, we estimate that the total radiation dose to a mouse from V/Q SPECT/CT is 321 mGy, well below the radiation dose required to have a significant negative impact on biological processes in BALB/c mice. This total dose could be reduced substantially if basic CT scans were acquired for co-registration purposes only instead of the multiple rotations required for high quality respiratory-gated images.

The characterisation of V/Q relationships in the lungs of healthy mice is a foundation upon which lung function can be studied under various physiological conditions and disease models. Respiratory diseases such as COPD would greatly benefit from V/Q imaging as our understanding of its progressive pathology and its impact on respiratory function could be considerably augmented by this lung function imaging methodology. We believe that the presented work demonstrates that we can reliably measure biomarkers of lung function using a clinically adapted method for performing V/Q SPECT in mice. The methodologies employed for delivery of radiolabelled tracer molecules, spatial alignment of images, and analysis of the resulting data provide a platform for investigations into the functional consequences of lung disease within experimental mouse models.

## References

[1] Bousquet J, Khaltaev N (2007) Global surveillance, prevention and control of chronic respiratory diseases: a comprehensive approach.: World Health Organization.

[2] Jones PW, Agusti AG (2006) Outcomes and markers in the assessment of chronic obstructive pulmonary disease. Eur Respir J 27: 822–832. 16585091 10.1183/09031936.06.00145104

[3] Petersson J, Glenny RW (2012) Imaging Regional PAO2 and Gas Exchange. J Appl Physiol. 10.1152/japplphysiol.00173.2012 22604886

[4] Wagner PD (2008) The multiple inert gas elimination technique (MIGET). Intensive Care Med 34: 994–1001. 18421437 10.1007/s00134-008-1108-6

[5] Miller MR, Quanjer PH, Swanney MP, Ruppel G, Enright PL (2011) Interpreting lung function data using 80% predicted and fixed thresholds misclassifies more than 20% of patients. Chest 139: 52–59. 20522571 10.1378/chest.10-0189

[6] Jogi J, Ekberg M, Jonson B, Bozovic G, Bajc M (2011) Ventilation/perfusion SPECT in chronic obstructive pulmonary disease: an evaluation by reference to symptoms, spirometric lung function and emphysema, as assessed with HRCT. Eur J Nucl Med Mol Imaging 38: 1344–1352. 21365251 10.1007/s00259-011-1757-5

[7] King GG, Harris B, Mahadev S (2010) V/Q SPECT: utility for investigation of pulmonary physiology. Semin Nucl Med 40: 467–473. 20920636 10.1053/j.semnuclmed.2010.07.006

[8] Suga K, Kawakami Y, Koike H, Iwanaga H, Tokuda O, et al (2010) Lung ventilation-perfusion imbalance in pulmonary emphysema: assessment with automated V/Q quotient SPECT. Ann Nucl Med 24: 269–277. 20340054 10.1007/s12149-010-0369-7

[9] Marsh S, Barnden L, O’Keeffe D (2011) Validation of co-registration of clinical lung ventilation and perfusion SPECT. Australas Phys Eng Sci Med 34: 63–68. 21331464 10.1007/s13246-011-0059-3

[10] Koba W, Kim K, Lipton ML, Jelicks L, Das B, et al (2011) Imaging devices for use in small animals. Semin Nucl Med 41: 151–165. 21440693 10.1053/j.semnuclmed.2010.12.003

[11] Franc BL, Acton PD, Mari C, Hasegawa BH (2008) Small-animal SPECT and SPECT/CT: important tools for preclinical investigation. J Nucl Med 49: 1651–1663. 18794275 10.2967/jnumed.108.055442

[12] Dolovich M, Labiris R (2004) Imaging drug delivery and drug responses in the lung. Proc Am Thorac Soc 1: 329–337. 16113454 10.1513/pats.200404-030MS

[13] Jobse BN, Johnson JR, Farncombe TH, Labiris R, Walker TD, et al (2009) Evaluation of allergic lung inflammation by computed tomography in a rat model in vivo. Eur Respir J 33: 1437–1447. 19164353 10.1183/09031936.00087508

[14] Froese AR, Ask K, Labiris R, Farncombe T, Warburton D, et al (2007) Three-dimensional computed tomography imaging in an animal model of emphysema. Eur Respir J 30: 1082–1089. 17804451 10.1183/09031936.00000507

[15] Mistry NN, Qi Y, Hedlund LW, Johnson GA (2010) Ventilation/perfusion imaging in a rat model of airway obstruction. Magn Reson Med 63: 728–735. 20146375 10.1002/mrm.22221 PMC2832088

[16] Togao O, Ohno Y, Dimitrov I, Hsia CC, Takahashi M (2011) Ventilation/perfusion imaging of the lung using ultra-short echo time (UTE) MRI in an animal model of pulmonary embolism. J Magn Reson Imaging. 10.1002/jmri.22645 PMC4573564 21761465

[17] Alfaro V, Roca-Acin J, Palacios L, Guitart R (2001) Multiple inert gas elimination technique for determining ventilation/perfusion distributions in rat during normoxia, hypoxia and hyperoxia. Clin Exp Pharmacol Physiol 28: 419–424. 11380516 10.1046/j.1440-1681.2001.03455.x

[18] Senden TJ, Moock KH, Gerald JF, Burch WM, Browitt RJ, et al (1997) The physical and chemical nature of technegas. J Nucl Med 38: 1327–1333. 9255177

[19] Maes F, Collignon A, Vandermeulen D, Marchal G, Suetens P (1997) Multimodality image registration by maximization of mutual information. IEEE Trans Med Imaging 16: 187–198. 9101328 10.1109/42.563664

[20] Press W, Teukolsky SA, Vetterling WT, Flannery BP (2007) (Powell’s) Methods in Multidimensions. Numerical Recipes in C: The Art of Scientific Computing. 3rd ed. Cambridge: Cambridge University Press. 509–515.

[21] Farncombe TH (2008) Software-based respiratory gating for small animal conebeam CT. Med Phys 35: 1785–1792. 18561653 10.1118/1.2905031

[22] Levitski MG (2007) Ventilation-Perfusion Relationships. In: Malley JEKG, editor. Pulmonary Physiology. 7th ed: McGraw-Hill. 113–129.

[23] West JB, Dollery CT (1960) Distribution of blood flow and ventilation-perfusion ratio in the lung, measured with radioactive carbon dioxide. J Appl Physiol 15: 405–410. 13844133 10.1152/jappl.1960.15.3.405

[24] Kreck TC, Krueger MA, Altemeier WA, Sinclair SE, Robertson HT, et al (2001) Determination of regional ventilation and perfusion in the lung using xenon and computed tomography. J Appl Physiol 91: 1741–1749. 11568158 10.1152/jappl.2001.91.4.1741

[25] Galvin I, Drummond GB, Nirmalan M (2007) Distribution of blood flow and ventilation in the lung: gravity is not the only factor. Br J Anaesth 98: 420–428. 17347182 10.1093/bja/aem036

[26] Sprung J, Gajic O, Warner DO (2006) Review article: age related alterations in respiratory function-anesthetic considerations. Can J Anaesth 53: 1244–1257. 17142659 10.1007/BF03021586

[27] Cardus J, Burgos F, Diaz O, Roca J, Barbera JA, et al (1997) Increase in pulmonary ventilation-perfusion inequality with age in healthy individuals. Am J Respir Crit Care Med 156: 648–653. 9279253 10.1164/ajrccm.156.2.9606016

[28] Holland J, Milic-Emili J, Macklem PT, Bates DV (1968) Regional distribution of pulmonary ventilation and perfusion in elderly subjects. J Clin Invest 47: 81–92. 16695948 10.1172/JCI105717 PMC297150

[29] Littleton SW (2012) Impact of obesity on respiratory function. Respirology 17: 43–49. 22040049 10.1111/j.1440-1843.2011.02096.x

[30] Fain SB, Altes TA, Panth SR, Evans MD, Waters B, et al (2005) Detection of age-dependent changes in healthy adult lungs with diffusion-weighted 3He MRI. Acad Radiol 12: 1385–1393. 16253850 10.1016/j.acra.2005.08.005

[31] Glenny RW, Lamm WJ, Albert RK, Robertson HT (1991) Gravity is a minor determinant of pulmonary blood flow distribution. J Appl Physiol 71: 620–629. 1938736 10.1152/jappl.1991.71.2.620

[32] Travis EL, Peters LJ, McNeill J, Thames HD Jr, Karolis C (1985) Effect of dose-rate on total body irradiation: lethality and pathologic findings. Radiother Oncol 4: 341–351. 3909241 10.1016/s0167-8140(85)80122-5

[33] Funk T, Sun M, Hasegawa BH (2004) Radiation dose estimate in small animal SPECT and PET. Med Phys 31: 2680–2686. 15487751 10.1118/1.1781553

